# Post Diagnosis Diet Quality and Colorectal Cancer Survival in Women

**DOI:** 10.1371/journal.pone.0115377

**Published:** 2014-12-15

**Authors:** Teresa T. Fung, Rutendo Kashambwa, Kaori Sato, Stephanie E. Chiuve, Charles S. Fuchs, Kana Wu, Edward Giovannucci, Shuji Ogino, Frank B. Hu, Jeffrey A. Meyerhardt

**Affiliations:** 1 Department of Nutrition, Simmons College, Boston, Massachusetts, United States of America; 2 Department of Neurology, Brigham and Women's Hospital, Boston, Massachusetts, United States of America; 3 Department of Nutrition, Harvard School of Public Health, Boston, Massachusetts, United States of America; 4 Channing Division of Network Medicine, Department of Surgery, Brigham and Women's Hospital, Harvard Medical School, Boston, Massachusetts, United States of America; 5 Department of Medicine, Brigham and Women's Hospital, Harvard Medical School, Boston, Massachusetts, United States of America; 6 Department of Medical Oncology, Dana Farber Cancer Institute, Boston, Massachusetts, United States of America; Tufts University, United States of America

## Abstract

**Background:**

Dietary factors are known to influence colorectal cancer (CRC) risk, however, their association with CRC survival is unclear. Therefore, we prospectively examined the association between diet quality scores, dietary patterns and colorectal cancer (CRC) survival.

**Methods:**

1201 women diagnosed with stage I–III CRC between 1986 and 2008, were followed through 2010. Diet was assessed via a food frequency questionnaire administered at least 6 months after diagnosis. We computed the Alternate Healthy Eating Index-2010 (AHEI-2010), alternate Mediterranean Diet score (aMED) and Dietary Approaches to Stop Hypertension score (DASH) and derived two dietary patterns, Western (unhealthy) and prudent (healthy), by principal component analysis for each woman.

**Results:**

During follow-up, we documented 435 deaths, including 162 from CRC. After adjusting for potential confounders, only a higher AHEI-2010 score was significantly associated with lower overall mortality (HR comparing extreme quintiles = 0.71, 95% CI 0.52–0.98, p trend = 0.01) as well as borderline significantly with lower risk of CRC mortality by the trend test (HR Q5 vs Q1 = 0.72, 95% CI = 0.43–1.21, p trend = 0.07). When AHEI-2010 components were examined separately, inverse associations for overall mortality were primarily accounted for by moderate alcohol intake (HR comparing abstainers vs 5–15 g/d = 1.30, 95%CI = 1.05–1.61) and lower intake of sugar sweetened beverages and fruit juices combined (HR for each additional serving = 1.11, 95% CI = 1.01–1.23). No other diet quality score or dietary pattern was associated with overall or CRC-specific mortality.

**Conclusion:**

Higher AHEI-2010 score may be associated with lower overall mortality, moderate alcohol consumption and lower consumption of sugar sweetened beverages and juices combined appeared to account for most of the observed associations.

## Introduction

Colorectal cancer (CRC) is the third leading cause of cancer death in women in the United States. It is also the third most common cancer in women [1] and has a moderate 5-year survival of slightly over 60% [2]. Among lifestyle factors, smoking after diagnosis may adversely influence survival [3] while higher levels of physical activity may have favorable influence [4].

Several studies have investigated overall diet quality and CRC incidence using predetermined scores such as the alternate Mediterranean (aMED) Diet Score and Dietary Approaches to Stop Hypertension (DASH) score. These scores are composed of various food groups and nutrients and award points for higher intake of healthy components and lower intake of unhealthy components; thus a higher score represents a healthier diet. Although they were not developed specifically for CRC prevention, studies found higher scores were associated with reduced CRC risk [5], [6]. Similarly, studies have also examined predominant empirical dietary patterns in the population and CRC risk. The two most common patterns that emerged were a “prudent/healthy” pattern characterized by higher intakes of fruits, vegetables, whole grains, and lean protein, and an “unhealthy/western” pattern characterized by high intakes of animal products, processed meats, and refined grains [7], [8]. Previous studies have shown an inverse association between the healthy/prudent and CRC risk and a direct association with Western pattern [7].

A healthy diet may improve survival after CRC diagnosis through reducing complications from cancer treatment or reducing inflammation [9], [10] or insulin secretion [11]. However, none of diet quality scores have been studied for survival among CRC patients. Only a small number of studies have examined the association between dietary patterns and CRC survival. In a U.S. study, the post-diagnosis Western pattern associated with shorter overall survival [12] and a study from Newfoundland province in Canada found that high adherence to the Processed Meat pattern, characterized by high intake of processed red meat and cured fish, before diagnosis, was associated with poorer overall survival among colon cancer survivors [13]. Studies on individual foods or nutrients also suggest that diet may influence survival. For example, red and processed meat [14] may be associated with shorter survival while higher pre-diagnostic serum folate levels may be associated with longer survival [15].

In the NHS, the DASH was previously associated with a lower risk of CRC [6] and the Alternate Healthy Eating Index-2010 (AHEI-2010) was associated with a lower overall cancer risk [16]. In addition, the Mediterranean diet has been shown to reduce CRC risk in other cohorts [17]. Therefore, in this analysis, we extend our research scope to prospectively investigate the association between three dietary quality scores, DASH, aMED, and the AHEI-2010 and two dietary patterns, the Western and Prudent patterns, measured after diagnosis, with colorectal cancer survival in women from the Nurses' Health Study (NHS). Results from this analysis may potentially allow clinicians to provide more guidance to patients to improve survival after CRC diagnosis.

## Methods

### Study Population

The NHS is a prospective cohort established in 1976 with 121,701 women registered nurses aged between 30 and 55 years at enrollment [18] [19]. A self-administered questionnaire was mailed to the study participants every 2 years to collect medical history, diet (every 4 years) and lifestyle information. This study was approved by the Institutional Review Board of the Brigham and Women's Hospital and all participants provided written informed consent. In addition, we obtained written permission from participants who were diagnosed with CRC to review their medical records to obtain information on tumor characteristics.

### Analytic population

We included women who were diagnosed with stages I to III colorectal cancer between 1986 and 2008. Colorectal cancer diagnosis was first self-reported by cohort participants in biennial questionnaires. Written permission was then sought from the patient to review medical records for confirmation by a physician. We extracted from medical records date of colorectal cancer diagnosis, tumor location, stage of disease, colon or rectal cancer histology, and tumor grade and data was de-identified before analysis. Individuals with a history of cancer within 3 years of colorectal cancer diagnosis and those who died within the first six months after the return of the first post-diagnosis biennial questionnaire or FFQ were excluded. After applying all exclusion criteria, a total of 1201 women were included in this analysis and followed through 2010.

### Mortality Ascertainment

Death was ascertained from state vital statistics records, the National Death Index, and through review of death certificates that were submitted via post by the deceased participants' next-of-kin. All data was de-identified before analysis. The follow-up for death was over 95% complete [20]. Overall survival was defined as the time from colorectal cancer diagnosis after return of the first post-diganosis FFQ to death by any cause. Colorectal cancer specific mortality was defined as the time from colorectal cancer diagnosis to death from colorectal cancer. Those who died without known tumor recurrence were included in the analysis of overall mortality but not the analysis of colorectal cancer mortality.

### Dietary Assessment

Diet was assessed by self-administered, semi-quantitative food frequency questionnaires (SFFQ) was in 1986, 1990, 1994, 1998, 2002 and 2006 [21]. Each FFQ contained approximately 130 items. A standard portion size and 9 possible frequency-of-consumption responses, ranging from “never, or less than once per month” to “6 or more times per day” were given for each food. Total energy and nutrient intake was calculated by summing up energy or nutrients from all foods [6], [12]. Prior validation studies showed reasonably good correlations between energy-adjusted nutrients assessed by the FFQ and multiple weeks of food records completed over the preceding year [22]. In this analysis, we used the first FFQ that was collected at least 6 months after diagnosis to minimize dietary intake affected by active treatment.

### Diet Quality Scores

The AHEI-2010 is updated from the original AHEI [23]. Components for this score were chosen based on their association with chronic diseases shown in the literature. The AHEI-2010 awards points for higher consumption of vegetables (excluding potatoes), whole fruit, whole grains, nuts and legumes, long chain omega-3 fatty acids, polyunsaturated fat; a lower consumption of sugar-sweetened beverages, red/processed meat, sodium, trans fat, and moderate alcohol consumption [16]. Each of these food groups has a range of 0 to 10 points, with a maximum overall score of 110 points.

The alternate Mediterranean Diet (aMED) was adapted from the Trichopolou score for the American population [24]. It awards 1 point for intake was greater than the cohort specific median in vegetables, legumes, fruits, nuts, whole grains, fish, and monounsaturated: saturated fat ratio; and one point if intake was less than the cohort median in meat, and if alcohol intake between 5 and 15 g/d for women [6]. The possible range for the aMED score was 0 to 9 points.

The DASH score was developed based on foods that are emphasized and discouraged in the Dietary Approaches to Stop Hypertension trial which was originally designed for blood pressure reduction [25]. It awards points for high intake of fruit, vegetables, nuts and legumes, low-fat dairy products, whole grains, low intake of red/processed meats, sweets, and sodium. For healthy food groups or nutrients, participants were award 1 point if they were in the lowest quintile, 2 point if they were in the next intake quintile, and those in the highest quintile are assigned 5 points. Scoring was reversed for unfavorable unhealthy food groups or nutrients [6].

### Dietary patterns

To identify predominant eating patterns in these cohorts, we grouped food items in FFQs into approximately 40 food groups and applied principal component analysis. This procedure utilizes correlations between food groups to derive factors (ie. dietary patterns) by generating linear combination of food groups that explains the variation of each derived pattern [26], [27]. The obtained factors were rotated by orthogonal transformation to derive patterns uncorrelated with each other and with more natural interpretation. We used the screen plot and eigen value (minimum of 1.5) to determine the number of patterns to retain. The prudent pattern was characterized by higher intakes of fruits, vegetables, whole grains, poultry, and low fat dairy products. The Western pattern was characterized by higher intakes of red and processed meats, refined grains, sweets and desserts, and high fat dairy products. Scores for each pattern was computed for each individual based on their intake of foods and the factor loadings of the foods (i.e., correlations with the patterns) and standardized to a mean of 0 with standard deviation = 1.

### Covariate Ascertainment

Height was assessed at inception of each cohort. Leisure time physical activity, body weight, and cigarette smoking was assessed in each biennial questionnaire. Weight change was calculated as the difference between the first weight reported on the biennial questionnaire at least 6 months after the diagnosis date and the last available weight reported in the biennial questionnaire before diagnosis.

### Statistical Analyses

Each of the first post-diagnosis diet quality scores and the dietary patterns was categorized into quintiles and diet was not updated during follow-up. We used Cox proportional hazard models to examine the association between post-diagnosis AHEI-2010, aMED, and DASH diet scores, the Western and Prudent dietary patterns and colorectal cancer survival and overall survival. Each diet quality score were modeled in separate regression models. Multivariable analyses were adjusted for covariates that may influence CRC survival. We included age (<50, 50–60, 61–70, 70+), physical activity (quintiles of metabolic equivalence task units per hours) [28], BMI (<21, 21 to 25, 25 to <30, 30+ kilograms per meters squared, and missing), weight change since diagnosis (lost more than 5 kg, −5 to 0, greater than 0 to 5, 5+) energy intake (continuous), smoking (never, past, current) stage of disease (I, II or III), grade of tumor differentiation (4 categories), tumor site (colon or rectal cancer), chemotherapy (yes/no) and year of diagnosis (5 categories). Alcohol (0, up to 10 g/d,>10 g/d) was adjusted only for the DASH score as it was a component of AHEI-2010 and aMed. Test of trend was conducted by fitting continuous terms for each of the diet quality scores or dietary pattern. The statistical analyses were conducted with SAS version 9.2 (SAS Institute, Cary, NC).

Because we observed a significant inverse association with the AHEI-2010 score, we examined consumption of food groups included in the AHEI-2010 in separate models to explore if the overall association of AHEI-2010 was driven by a few components. For each model we additionally adjusted the analysis by a modified AHEI-2010 that does not have that particular component.

## Results

During follow-up from 1986 to 2010, among 1201 women, 435 died, including 162 from colorectal cancer. The median follow-up was 11.2 years and median survival was 8.0 years (Table 1). 79.4% of the tumors were in the colon. The median time from diagnosis to return of the FFQ was 21.0 months. The median age at entry to analysis was 66.5 y, median BMI was 25.4 kg/m^2^, and 8.9% participants were current smokers. The AHEI-2010 was moderately correlated with aMed (Spearman r = 0.53, p<0.0001) and the DASH score (Spearman r = 0.57, p<0.0001).

**Table 1:** Health and lifestyle characteristics at entry to analysis (n = 1201).

Participant characteristics	
Age at diagnosis (years), median (IQR)	66.5 (60.9–72.2)
Survival (years), median (IQR)	8.0 (3.9–12.0)
Year of diagnosis (n)	
January 1986–December 1989	191 (15.9%)
January 1990–December 1994	242 (20.1%)
January 1995–December 1999	327 (27.2%)
January 2000–June 2010	441 (36.7%)
Site (n)	
Colon	953 (79.4%)
Rectum	247 (20.6%)
Stage (n)	
I	317 (26.4%)
II	366 (30.5%)
III	282 (23.5%)
missing	236 (19.6%)
Tumor differential grade (n)	
Well	155 (12.9%)
Moderate	657 (54.7%)
Poor/undifferentiated	145 (12.1%)
unknown	244 (20.3%)
BMI (kg/m2), median (IQR)	25.4 (22.7–29.2)
Physical Activity (METs/week*), median (IQR)	7.9 (2.7–20.3)
Current Smoker (%)	8.9
Energy intake post-diagnosis (kcal/day) median (IQR)	1624 (1293–2041)
Alcohol intake post-diagnosis (g/day) median (IQR)	0 (0–5.5)
Total Calcium intake (mg/day) median (IQR)	1097 (724–1576)
Total Fiber intake (g/day) median (IQR)	19 (16–23)
Total Folate intake (mcg/day) median (IQR)	393 (289–630)

*Metabolic Equivalent hours.

After adjusting for potential confounders, we observed a lower overall mortality for higher AHEI-2010 score (HR for AHEI-2010 score Q5 vs Q1 = 0.71, 95% CI 0.52–0.98, p trend  = 0.01) (Table 2). No other diet quality scores or dietary patterns were associated with overall survival after multivariable adjustment. Additional adjustment for pre-diagnosis diet quality scores or dietary pattern scores did not change the results. For example, HR for AHEI-2010 comparing Q5 and Q1 = 0.69 (95% CI = 0.50–1.6, p trend = 0.001). For CRC-specific mortality, there was no association with any of the diet quality scores or dietary patterns although results for the AHEI-2010 was borderline significant for the trend test (Q5 vs Q1 = 0.72 95% CI 0.43–1.21, p trend  = 0.07) (Table 3). Additional analysis excluding survivors who died within one year of diagnosis did not materially change the results (S1 Table).

**Table 2:** Association between quintiles of post-diagnosis diet score (Hazard ratio and 95% CI) and overall mortality.

	Q1	Q2	Q3	Q4	Q5	P trend
**AHEI score**						
Median score	39	47	53	58	68	
Number of cases	116	95	86	72	66	
Age & energy adjusted	1.00	0.76 (0.58, 1.00)	0.65 (0.49, 0.86)	0.59 (0.44, 0.80)	0.57 (0.42, 0.77)	<0.0001
Multivariate* adjusted	1.00	0.84 (0.63, 1.10)	0.71 (0.53, 0.94)	0.71 (0.52, 0.96)	0.71 (0.52, 0.98)	0.01
**aMED score**						
Median score	2	3	4	5	6	
Number of cases	113	83	83	68	88	
Age & energy adjusted	1.00	1.02 (0.77, 1.36)	0.82 (0.61, 1.09)	0.65 (0.48, 0.88)	0.59 (0.43, 0.79)	<0.0001
Multivariate* adjusted	1.00	1.14 (0.85, 1.52)	1.01 (0.75, 1.37)	0.92 (0.66, 1.27)	0.87 (0.63, 1.21)	0.31
**DASH score**						
Median score	17	21	23	26	30	
Number of cases	103	92	66	91	83	
Age & energy adjusted	1.00	0.79 (0.60, 1.05)	0.75 (0.55, 1.02)	0.69 (0.52, 0.92)	0.68 (0.50, 0.91)	0.003
Multivariate* adjusted	1.00	0.92 (0.68, 1.24)	0.96 (0.69, 1.32)	0.87 (0.65, 1.18)	0.98 (0.71, 1.35)	0.66
**PRUDENT pattern**						
Median score	−1.1	−0.6	−0.2	0.4	1.3	
Number of cases	104	78	86	89	78	
Age and energy adjusted	1.00	0.67 (0.50, 0.89)	0.71 (0.53, 0.95)	0.67 (0.49, 0.91)	0.59 (0.42, 0.82)	0.007
Multivariate * adjusted	1.00	0.84 (0.62, 1.13)	0.91 (0.67, 1.25)	1.02 (0.73, 1.42)	0.93 (0.65, 1.34)	0.80
**WESTERN pattern**						
Median score	−1.1	−0.6	−0.1	0.4	1.3	
Number of cases	71	85	84	98	97	
Age and energy adjusted	1.00	1.24 (0.91, 1.71)	1.01 (0.73, 1.40)	1.48 (1.07, 2.06)	1.46 (1.01, 2.11)	0.03
Multivariate adjusted	1.00	1.15 (0.83, 1.58)	1.02 (0.72, 1.43)	1.37 (0.97, 1.94)	1.32 (0.89, 1.97)	0.23

*Adjusted for age, physical activity, BMI, weight change, cancer grade, chemotherapy, smoking status, energy intake, colon or rectal cancer, stage of disease, and date of colorectal cancer diagnosis.

**Table 3:** Association between quintiles of post-diagnosis diet score (Hazard ratio and 95% CI) and colorectal cancer mortality.

	Q1	Q2	Q3	Q4	Q5	P trend
**AHEI score**						
Number of cases	44	28	34	30	26	
Age & energy adjusted	1.00	0.61 (0.38, 0.98)	0.72 (0.46, 1.13)	0.67 (0.42, 1.06)	0.58 (0.36, 0.94)	0.02
Multivariate* adjusted	1.00	0.69 (0.42, 1.12)	0.73 (0.45, 1.17)	0.76 (0.47, 1.23)	0.72 (0.43, 1.21)	0.07
**aMED score**						
Number of cases	39	32	32	23	36	
Age & energy adjusted	1.00	1.02 (0.64, 1.63)	0.89 (0.56, 1.44)	0.63 (0.37, 1.07)	0.64 (0.40, 1.05)	0.02
Multivariate* adjusted	1.00	1.18 (0.73, 1.91)	0.96 (0.58, 1.56)	0.73 (0.42, 1.28)	0.84 (0.50, 1.42)	0.19
**DASH score**						
Number of cases	40	37	21	30	34	
Age & energy adjusted	1.00	0.80 (0.51, 1.26)	0.61 (0.36, 1.03)	0.61 (0.38, 0.98)	0.72 (0.45, 1.13)	0.06
Multivariate* adjusted	1.00	0.84 (0.52, 1.34)	0.70 (0.41, 1.22)	0.72 (0.43, 1.20)	0.87 (0.52, 1.45)	0.35
**PRUDENT pattern**						
Number of cases	39	26	30	35	32	
Age & energy adjusted	1.00	0.59 (0.36, 0.98)	0.66 (0.40, 1.07)	0.70 (0.43, 1.14)	0.59 (0.34, 1.00)	0.04
Multivariate* adjusted	1.00	0.67 (0.40, 1.12)	0.62 (0.37, 1.05)	0.91 (0.53, 1.55)	0.67 (0.37, 1.22)	0.16
**WESTERN pattern**						
Number of cases	23	36	25	38	40	
Age & energy adjusted	1.00	1.56 (0.92, 2.64)	0.99 (.056, 1.77)	1.63 (0.93, 2.83)	1.73 (0.94, 3.20)	0.08
Multivariate* adjusted	1.00	1.48 (0.87, 2.54)	1.00 (0.55, 1.83)	1.50 (0.84, 2.70)	1.66 (0.85, 3.23)	0.09

*Adjusted for age, physical activity, BMI, weight change, cancer grade, chemotherapy, smoking status, energy intake, colon or rectal cancer, stage of disease, and date of colorectal cancer diagnosis.

We then examined each food group included in the AHEI-2010. Compared with women consuming 5–15 g of alcohol per day, a higher risk for overall mortality was found for non-consumers (HR = 1.30, 95% CI = 1.05–1.61) (Table 4). The AHEI-2010 includes a component that combines sugar sweetened beverages and fruit juices and awards points for lower consumption. We observed a higher overall mortality with this food group (HR for each serving per day = 1.11, 95% CI = 1.01–1.23). For CRC-specific mortality, we observed a lower risk for each daily serving of nuts consumption (HR = 0.69, 95% CI = 0.49–0.97). These associations remained essentially unchanged and statistically significant after adjusting for a version of the AHEI-2010 score that did not include the particular component.

**Table 4:** Multivariable* hazard ratio (95% CI) of AHEI-2010 components (per serving per day unless otherwise specified) for mortality in NHS (1986–2010).

Components	Overall mortality	Colorectal cancer mortality
Red/processed meat	1.07 (0.87, 1.30)	1.22 (0.90, 1.67)
Nuts	0.98 (0.82, 1.17)	0.69 (0.49, 0.97)**
Sugar sweetened beverages + juices	1.11 (1.01, 1.23)**	1.16 (0.99, 1.35)
Whole grains	0.98 (0.95, 1.01)	0.97 (0.93, 1.02)
Vegetables	1.00 (0.94, 1.06)	0.94 (0.84, 1.04)
Whole fruits	1.08 (0.98, 1.20)	1.03 (0.87, 1.21)
Polyunsaturated to saturated fat ratio	0.98 (0.93, 1.03)	1.00 (0.93, 1.07)
Long chain omega-3	0.87 (0.53, 1.43)	0.63 (0.26, 1.54)
Sodium	1.00 (0.95, 1.05)	0.94 (0.86, 1.03)
Trans fat	0.97 (0.88, 1.07)	1.07 (0.92, 1.24)
Alcohol		
No intake	1.30 (1.05, 1.61)**	1.32 (0.93, 1.87)
5–15 g/day	1 (ref)	1 (ref)
>15 g/day	1.22 (0.85, 1.76)	0.97 (0.50, 1.87)

*Adjusted for age, physical activity, BMI, weight change, cancer grade, chemotherapy, smoking status, energy intake, colon or rectal cancer, stage of disease, date of colorectal cancer diagnosis.

**After additional adjustment for an AHEI-2010 score without the specific component, RR of overall mortality for alcohol, sweetened beverages + juices remained unchanged and significant, but for nuts and CRC mortality was slightly attenuated and no longer significant (RR = 0.71, 95% CI = 0.50 = 1.01).

## Discussion

In this analysis, we found the AHEI-2010 score was associated with longer overall survival among CRC survivors. Among components of AHEI2010, non-consumption of alcohol and higher consumption of combined sugar sweetened beverages and fruit juices may contribute to the poorer survival in women. None of the other diet quality scores examined in this analysis was associated with overall or CRC mortality.

The AHEI-2010 emphasize a high intake of fruits, vegetables, whole grains, legumes and nuts and decreased intake of salt and saturated fat [6], [29]. A recent study in the NHS and HPFS showed that higher diet scores for the AHEI-2010 are associated with decreased risk for major chronic disease, including cancer [16]. A search of the literature in the past 10 years did not find any studies on diet quality scores and CRC survival. A small number of studies focused on specific nutrients with mixed results for pre- and post-diagnosis serum folate and survival [15], [30] and no association for serum carotenoids [31]. In a small study with 36 colon cancer patients, flavonoids supplement reduced colorectal cancer recurrence [32].

Although the different dietary quality scores all emphasized higher fruits and vegetables intake and less red and processed meat intake, only the AHEI-2010 showed a significant inverse association with overall mortality. This association may partly be due to the AHEI-2010 having a maximum possible score of 100, while DASH and aMED have a much narrower possible score range, and therefore may not be able to finely discriminate dietary healthfulness. In addition, components that differ between the diet quality scores may contribute to the association. In our study, we found that lower consumption of sugar sweetened beverages, a component included only in the AHEI-2010 was associated with lower overall mortality. In a previous study in men, we found sugar sweetened beverage consumption was associated with higher CRC risk [6]. Sugar sweetened beverages has been associated with markers of insulin resistance [33], and elevated glucose or diabetes has been associated with poorer survival in CRC patients [34], [35]. Inflammation has been suggested as an unfavorable indicator for survival among colorectal cancer survivors [9], [10] and in the general population [36]. Previous data from the Nurses' Health Study shown that the original version of the AHEI and the aMed were inversely associated with inflammatory markers [37]. This may partly mediate our observation on the AHEI-2010 and overall survival. The prudent and Western patterns reflect predominant eating patterns in our cohort. Therefore, they may include food groups that are not associated with survival.

Few studies have examined dietary patterns and CRC survival. In an adjuvant therapy trial (CALGB 89803), similar dietary patterns as our cohorts were derived and the Western diet score was associated with a higher mortality among patients with stage III colon cancer [12]. A study from Newfoundland found the pre-diagnosis processed meat pattern, characterized by higher intakes of red and processed meat, cured and non-cured fish, resulted in shorter overall survival for individuals at the highest tertile of the score compared to those at the bottom tertile [13]. In addition, the U.S. Cancer Prevention Study II Nutrition cohort showed higher red meat intake before diagnosis was associated with worse survival [14]. On the other hand, we did not observe any significant association with the Western pattern. Our study included only a small number of deaths and the power was limited, however there was a suggestion of a direct association for both overall and CRC mortality.

Strengths of our study include comprehensive information on relevant potential confounders. In addition, we have detailed diet data which allowed us to compute and compare different diet scores to assess potential association between survival and overall diet quality. Some of the diet scores, especially the DASH score, was primarily designed for cardiovascular disease prevention and may not have included food components important for CRC survival. However, since only about half of the deaths in this study were from CRC, it is still important to view the diets represented by these scores from an overall health promotion perspective. Although the FFQ is validated, there is still a certain degree of error because of its self-report nature. Although we have a substantial number of deaths, the causes of death were quite varied. The non-CRC deaths were from CVD, non-CRC neoplasms, respiratory, and other deaths. The case numbers for each cause of the non-CRC deaths is too small to separately examine each of these causes. However, studies have shown a diet high in fruits and vegetables, whole grains, and low in red/processed meats, refined grains is associated with lower mortality [38]–[40]. Although we have adjusted for potential confounders extensively, these were self-reported and residual confounding cannot be completely avoided. Also, the women in our cohort were mostly white and well-educated; therefore our results need to be replicated in other populations and also in men.

## Conclusions

We found that a higher AHEI-2010 score may be associated with lower overall mortality, inverse associations were primarily explained by moderate intake of alcohol and lower intake of sugar sweetened beverages and juices combined.

## Supporting Information

S1 Table **Hazard ratio (95% CI) for overall mortality by quintiles of post-diagnosis diet score.**
(DOCX)

## References

[1] Colorectal Cancer Statistics. Centers for Disease Control and Prevention. Available: http://www.cdc.gov/cancer/colorectal/statistics/, Accessed 2013 Jan 28.

[2] Lofano K, Principi M, Scavo MP, Pricci M, Ierardi E, et al. (2012) Dietary Lifestyle and Colorectal Cancer Onset, Recurrence, and Survival: Myth or Reality? Journal of Gastrointestinal Cancer: 1–11. 10.1007/s12029-012-9425-y 22878898

[3] Walter V, Jansen L, Hoffmeister M, Brenner H (2014) Smoking and survival of colorectal cancer patients: systematic review and meta-analysis. Annals of Oncology 25:1517–1525. 24692581 10.1093/annonc/mdu040

[4] Meyerhardt JA, Heseltine D, Niedzwiecki D, Hollis D, Saltz LB, et al (2006) Impact of physical activity on cancer recurrence and survival in patients with stage III colon cancer: findings from CALGB 89803. Journal of Clinical Oncology 24:3535–3541. 16822843 10.1200/JCO.2006.06.0863

[5] Reedy J, Mitrou PN, Krebs-Smith SM, Wirfalt E, Flood A, et al (2008) Index-based dietary patterns and risk of colorectal cancer: the NIH-AARP Diet and Health Study. Am J Epidemiol 168:38–48. 18525082 10.1093/aje/kwn097 PMC6287243

[6] Fung TT, Hu FB, Wu K, Chiuve SE, Fuchs CS, et al (2010) The Mediterranean and Dietary Approaches to Stop Hypertension (DASH) diets and colorectal cancer. Am J Clin Nutr 92:1429–1435. 21097651 10.3945/ajcn.2010.29242 PMC2980967

[7] Fung TT, Brown LS (2013) Dietary patterns and risk of colorectal cancer. Current Nutrition Reports 2:48–55. 24496398 10.1007/s13668-012-0031-1 PMC3649851

[8] Randi G, Edefont V, Ferraroni M, La Vecchia C, Decarli A (2010) Dietary patterns and the risk of colorectal cancer and adenomas. Nutrition Reviews 68:389–408. 20591107 10.1111/j.1753-4887.2010.00299.x

[9] Cooney RV, Chai W, Franke AA, Wilkens LR, Kolonel LN, et al (2013) C-reactive protein, lipid-soluble micronutrients, and survival in colorectal cancer patients. Cancer Epidemiol Biomarkers & Prevention 22:1278–1288. 10.1158/1055-9965.EPI-13-0199 PMC3834261 23677577

[10] Crozier JE, McKee RF, McArdle CS, Angerson WJ, Anderson JH, et al (2006) The presence of a systemic inflammatory response predicts poorer survival in patients receiving adjuvant 5-FU chemotherapy following potentially curative resection for colorectal cancer. British Journal of Cancer 94:1833–1836. 16721360 10.1038/sj.bjc.6603185 PMC2361334

[11] Wolpin BM, Meyerhardt JA, Chan AT, Ng K, Chan JA, et al (2009) Insulin, the insulin-like growth factor axis, and mortality in patients with nonmetastatic colorectal cancer. Journal of Clinical Oncology 27:176–185. 19064975 10.1200/JCO.2008.17.9945 PMC2645084

[12] Meyerhardt JA, Niedzwiecki D, Hollis D, Saltz LB, Hu FB, et al (2007) Association of dietary patterns with cancer recurrence and survival in patients with stage III colon cancer. JAMA: the journal of the American Medical Association 298:754–764. 17699009 10.1001/jama.298.7.754

[13] Zhu Y, Wu H, Wang PP, Savas S, Woodrow J, et al. (2013) Dietary patterns and colorectal cancer recurrence and survival: a cohort study. BMJ Open 3: pii: e002270. 10.1136/bmjopen-2012-002270 PMC3586110 23396503

[14] McCullough ML, Gapstur SM, Shah R, Jacobs EJ, Campbell PT (2013) Association between red and processed meat intake and mortality among colorectal cancer survivors. Journal of Clinical Oncology 31:2773–2782. 23816965 10.1200/JCO.2013.49.1126 PMC3718877

[15] Wolpin BM, Wei EK, Ng K, Meyerhardt JA, Chan JA, et al (2008) Prediagnostic plasma folate and the risk of death in patients with colorectal cancer. Journal of Clinical Oncology 26:3222–3228. 18591557 10.1200/JCO.2008.16.1943 PMC3962289

[16] Chiuve SE, Fung TT, Rimm EB, Hu FB, McCullough ML, et al (2012) Alternative dietary indices both strongly predict risk of chronic disease. J Nutr 142:1009–1018. 22513989 10.3945/jn.111.157222 PMC3738221

[17] Schwingshackl L, Hoffmann G (2014) Adherence to Mediterranean diet and risk of cancer: A systematic review and meta-analysis of observational studies. International Journal of Cancer 135:1884–1897. 24599882 10.1002/ijc.28824

[18] Colditz GA, Martin P, Stampfer MJ, Willett WC, Sampson L, et al (1986) Validation of questionnaire information on risk factors and disease outcomes in a prospective cohort study of women. Am J Epidemiol 123:894–900. 3962971 10.1093/oxfordjournals.aje.a114319

[19] Hennekens C, Speizer F, Rosner B, Bain C, Belanger C, et al (1979) Use Of Permanent Hair Dyes And Cancer Among Registered Nurses. The Lancet 313:1390–1393. 10.1016/s0140-6736(79)92021-x 87844

[20] Rich-Edwards JW, Corsano KA, Stampfer MJ (1994) Test of the National Death Index and Equifax nationwide death search. Am J Epidemiol 140:1016–1019. 7985649 10.1093/oxfordjournals.aje.a117191

[21] Willett WC (1998) Nutritional Epidemiology. New York, NY: Oxford University Press.

[22] Rimm EB, Giovannucci EL, Stampfer MJ, Colditz GA, Litin LB, et al (1992) Reproducibility and validity of an expanded self-administered semiquantitative food frequency questionnaire among male health professionals. Am J Epidemiol 135:1114–1126. 1632423 10.1093/oxfordjournals.aje.a116211

[23] McCullough ML, Feskanich D, Stampfer MJ, Giovannucci EL, Rimm EB, et al (2002) Diet quality and major chronic disease risk in men and women: moving toward improved dietary guidance. Am J Clin Nutr 76:1261–1271. 12450892 10.1093/ajcn/76.6.1261

[24] Trichopoulou A, Costacou T, Bamia C, Trichopoulos D (2003) Adherence to a Mediterranean diet and survival in a Greek population. N Engl J Med 348:2599–2608. 12826634 10.1056/NEJMoa025039

[25] Sacks FM, Svetkey LP, Vollmer WM, Appel LJ, Bray GA, et al (2001) Effects on blood pressure of reduced dietary sodium and the Dietary Approaches to Stop Hypertension (DASH) diet. DASH-Sodium Collaborative Research Group. New England Journal of Medicine 344:3–10. 11136953 10.1056/NEJM200101043440101

[26] Hu FB, Rimm E, Smith-Warner SA, Feskanich D, Stampfer MJ, et al (1999) Reproducibility and validity of dietary patterns assessed with a food-frequency questionnaire. Am J Clin Nutr 69:243–249. 9989687 10.1093/ajcn/69.2.243

[27] Fung TT, Willett WC, Stampfer MJ, Manson JE, Hu FB (2001) Dietary patterns and the risk of coronary heart disease in women. Archives of Internal Medicine 161:1857. 11493127 10.1001/archinte.161.15.1857

[28] Ainsworth BE, Haskell WL, Whitt MC, Irwin ML, Swartz AM, et al (2000) Compendium of physical activities: an update of activity codes and MET intensities. Medicine and science in sports and exercise 32:498–504. 10.1097/00005768-200009001-00009 10993420

[29] Kim EH, Willett WC, Fung T, Rosner B, Holmes MD (2011) Diet quality indices and postmenopausal breast cancer survival. Nutr Cancer 63:381–388. 21462090 10.1080/01635581.2011.535963 PMC3113520

[30] Byström P, Björkegren K, Larsson A, Johansson L, Berglund Å (2009) Serum vitamin B12 and folate status among patients with chemotherapy treatment for advanced colorectal cancer. Upsala journal of medical sciences 114:160–164. 19736606 10.1080/03009730903027172 PMC2852767

[31] Leung EY, Crozier JE, Talwar D, O'Reilly DSJ, McKee RF, et al (2008) Vitamin antioxidants, lipid peroxidation, tumour stage, the systemic inflammatory response and survival in patients with colorectal cancer. International Journal of Cancer 123:2460–2464. 18729200 10.1002/ijc.23811

[32] Hoensch H, Groh B, Edler L, Kirch W (2008) Prospective cohort comparison of flavonoid treatment in patients with resected colorectal cancer to prevent recurrence. World Journal of Gastroenterology 14:2187. 18407592 10.3748/wjg.14.2187 PMC2703843

[33] Yoshida M, McKeown NM, Rogers G, Meigs JB, Saltzman E, et al (2007) Surrogate markers of insulin resistance are associated with consumption of sugar-sweetened drinks and fruit juice in middle and older-aged adults. Journal of Nutrition 137:2121–2127. 17709452 10.1093/jn/137.9.2121

[34] Dehal AN, Newton CC, Jacobs EJ, Patel AV, Gapstur SM, et al (2012) Impact of diabetes mellitus and insulin use on survival after colorectal cancer diagnosis: the Cancer Prevention Study-II Nutrition Cohort. Journal of Clinical Oncology 30:53–59. 22124092 10.1200/JCO.2011.38.0303

[35] Yang Y, Mauldin PD, Ebeling M, Hulsey TC, Liu B, et al (2013) Effect of metabolic syndrome and its components on recurrence and survival in colon cancer patients. Cancer 119:1512–1520. 23280333 10.1002/cncr.27923

[36] Ahmadi-Abhari S, Luben R, Wareham N, Khaw K-T (2013) Seventeen year risk of all-cause and cause-specific mortality associated with C-reactive protein, fibrinogen and leukocyte count in men and women: the EPIC-Norfolk study. European Journal of Epidemiology 28:541–550. 23821244 10.1007/s10654-013-9819-6

[37] Fung TT, McCullough ML, Newby P, Manson JE, Meigs JB, et al (2005) Diet-quality scores and plasma concentrations of markers of inflammation and endothelial dysfunction. Am J Clin Nutr 82:163–173. 16002815 10.1093/ajcn.82.1.163

[38] Fung TT, Rexrode KM, Mantzoros CS, Manson JE, Willett WC, et al (2009) Mediterranean diet and incidence of and mortality from coronary heart disease and stroke in women. Circulation 119:1093–1100. 19221219 10.1161/CIRCULATIONAHA.108.816736 PMC2724471

[39] Mitrou PN, Kipnis V, Thiebaut A, Reedy J, Subar AF, et al (2007) Mediterranean dietary pattern and prediction of all-cause mortality in a US population: results from the NIH-AARP Diet and Health Study. Archives of Internal Medicine 167:2461. 18071168 10.1001/archinte.167.22.2461

[40] Knoops KT, de Groot LC, Kromhout D, Perrin A-E, Moreiras-Varela O, et al (2004) Mediterranean diet, lifestyle factors, and 10-year mortality in elderly European men and women. JAMA: the journal of the American Medical Association 292:1433–1439. 15383513 10.1001/jama.292.12.1433

