# Supplementary material for: Post Diagnosis Diet Quality and Colorectal Cancer Survival in Women
Source: PLoS One. 2014 Dec 15;9(12):e115377. doi: 10.1371/journal.pone.0115377 (PMC4266679; doi:10.1371/journal.pone.0115377)
Supplement: S1 Table — Hazard ratio (95% CI) for overall mortality by quintiles of post-diagnosis diet score. (DOCX) [file pone.0115377.s001.docx]

Supplemental table S1: Hazard ratio (95% CI) for overall mortality by quintiles of post-diagnosis diet score

|  | **Q1** | **Q2** | **Q3** | **Q4** | **Q5** | **P trend** |
| --- | --- | --- | --- | --- | --- | --- |
|  |  |  |  |  |  |  |
| **AHEI score** |  |  |  |  |  |  |
| Median score | 39 | 47 | 53 | 58 | 68 |  |
| Number of cases | 108 | 90 | 79 | 65 | 61 |  |
| Age & energy adjusted | 1.00 | 0.79 (0.60, 1.04) | 0.64 (0.48, 0.86) | 0.57 (0.42, 0.78) | 0.58 (0.42, 0.79) | <0.0001 |
| Multivariate* adjusted | 1.00 | 0.87 (0.65, 1.15) | 0.69 (0.52, 0.94) | 0.68 (0.50, 0.94) | 0.71 (0.51, 1.00) | 0.009 |
|  |  |  |  |  |  |  |
| **aMED score** |  |  |  |  |  |  |
| Median score | 2 | 3 | 4 | 5 | 6 |  |
| Number of cases | 104 | 77 | 73 | 64 | 85 |  |
| Age & energy adjusted | 1.00 | 1.04 (0.77, 1.40) | 0.78 (0.57, 1.06) | 0.65 (0.47, 0.90) | 0.61 (0.45, 0.83) | 0.0001 |
| Multivariate* adjusted | 1.00 | 1.16 (0.86, 1.58) | 0.95 (0.69, 1.30) | 0.89 (0.63, 1.25) | 0.88 (0.63, 1.23) | 0.30 |
|  |  |  |  |  |  |  |
| **DASH score** |  |  |  |  |  |  |
| Median score | 17 | 21 | 23 | 26 | 30 |  |
| Number of cases | 94 | 83 | 64 | 83 | 79 |  |
| Age & energy adjusted | 1.00 | 0.78 (0.58, 1.05) | 0.79 (0.58, 1.09) | 0.68 (0.51, 0.92) | 0.70 (0.52, 0.95) | 0.005 |
| Multivariate* adjusted | 1.00 | 0.90 (0.66, 1.23) | 1.01 (0.72, 1.40) | 0.85 (0.62, 1.17) | 1.00 (0.71, 1.40) | 0.69 |
|  |  |  |  |  |  |  |
| **PRUDENT pattern** |  |  |  |  |  |  |
| Median score | -1.1 | -0.6 | -0.2 | 0.4 | 1.3 |  |
| Number of cases | 94 | 74 | 77 | 85 | 73 |  |
| Age and energy adjusted | 1.00 | 0.69 (0.51, 0.94) | 0.72 (0.53, 0.97) | 0.71 (0.52, 0.98) | 0.61 (0.43, 0.86) | 0.02 |
| Multivariate * adjusted | 1.00 | 0.88 (0.64, 1.20) | 0.91 (0.66, 1.27) | 1.07 (0.76, 1.50) | 0.95 (0.65, 1.39) | 0.90 |
|  |  |  |  |  |  |  |
| **WESTERN pattern** |  |  |  |  |  |  |
| Median score | -1.1 | -0.6 | -0.1 | 0.4 | 1.3 |  |
| Number of cases | 64 | 77 | 80 | 91 | 91 |  |
| Age and energy adjusted | 1.00 | 1.24 (0.89, 1.74) | 1.06 (0.75, 1.48) | 1.51 (1.07, 2.13) | 1.49 (1.02, 2.20) | 0.03 |
| Multivariate adjusted | 1.00 | 1.15 (0.82, 1.61) | 1.04 (0.73, 1.47) | 1.39 (0.97, 2.00) | 1.32 (0.87, 1.99) | 0.28 |
|  |  |  |  |  |  |  |

*Adjusted for age, physical activity, BMI, weight change, cancer grade, chemotherapy, smoking status, energy intake, colon or rectal cancer, stage of disease, and date of colorectal cancer diagnosis
